# Hypophysectomy, pituitary neuroadenolysis and pituitary radiosurgery for the treatment of refractory cancer pain: a historical review and mechanism investigation

**DOI:** 10.3389/fneur.2024.1529944

**Published:** 2025-01-10

**Authors:** Yuchen Hu, Wanghao Zhang, Zijian Chen, Xiaoyan Wu, Shuaishuai Xue, Yangqi Mao, Peiyao Yi, Jiezuo Wei, Dadi Qian, Xingqin Wang, Peidong Zhang, Hao Long

**Affiliations:** ^1^Department of Neurosurgery, Nanfang Hospital, Southern Medical University, Guangzhou, China; ^2^Institute of Brain Diseases, Nanfang Hospital, Southern Medical University, Guangzhou, China; ^3^The First Clinical Medical College, Southern Medical University, Guangzhou, China

**Keywords:** analgesic effects, cancer pain, gamma knife radiosurgery, hypophysectomy, pituitary, pain management

## Abstract

Refractory cancer pain affects 10–20% of patients with advanced malignancies and is not adequately controlled by opioids. The intrathecal therapy is an effective interventional procedure for referral, but the implanted infusion pumps are costly and the refilling requires technical expertise. Hypophysectomy, in its three stages—surgical, chemical, and radiosurgical—has emerged as an alternative for managing this pain. However, the underlying mechanism remains elusive, with existing hypotheses unable to comprehensively account for both the initial and long-term analgesic effects. This literature review explores the historical evolution, clinical outcomes, and hypothesized mechanisms of hypophysectomy for pain relief. Surgical hypophysectomy initially demonstrated an 85.5% success rate but carried significant risks like diabetes insipidus and hypopituitarism. Chemical hypophysectomy reduced invasiveness, achieving 75.1% pain relief with fewer complications. Modern pituitary radiosurgery has improved safety while maintaining high efficacy (initial relief: 95.9%, long-term: 73.5%). The mechanisms underlying pain relief remain unclear but include tumor regression, increased *β*-endorphins, neuroendocrine modulation, and hypothalamic involvement. A new hypothesis suggests that radiosurgery induces hormone redistribution (e.g., oxytocin, vasopressin) through hypothalamic–pituitary modulation, contributing to both immediate and long-term analgesia. Despite its potential, unresolved issues such as optimal radiation dose, pain assessment standardization, and precise mechanisms limit widespread adoption. This review underscores the need for larger, homogenous studies to validate the safety and efficacy of hypophysectomy in treating refractory cancer pain. These findings offer a promising avenue for improving palliative care in oncology.

## 1 Introduction

Severe pain occurs in approximately 80% of the patients with advanced-stage cancer with a variety of other symptoms, including fatigue, anorexia, cachexia, chronic nausea, dyspnea, anxiety, and depression (1, 2). Refractory cancer pain, not responsive to standard treatment with opioids and co-analgesics of at least 3 months duration, occurs in 10–20% of patients (3, 4). Pain management is crucial for adequate palliation of both physical and psychological symptoms to improve quality of life.

Opioids, the principal analgesics for severe pain, have high risks of addiction, tolerance, drug poisoning, and even overdose deaths. Clinical guidelines suggest that the failure to achieve adequate analgesia and the presence of intolerable adverse effects should be major indications for referral from opioids to interventional strategies. However, there is a lack of formal guidelines. The intrathecal therapy(also called intrathecal drug delivery system, IDDS) is the most widely-used procedure for referral, but the pumps are costly and require frequent device revisions (5, 6). The annual rate for IDDS complications requiring surgical intervention is 10.5% (1). Other interventional consultations including percutaneous vertebral augmentation, cementoplasty, neurodestructive procedures using spinal analgesics are mostly for specific syndromes such as bone lesions (6).

Hypophysectomy was carried out to produce objective regression of metastatic hormone-sensitive cancer in the first place, but was noted to have a consistent pain relief effect. The preliminary reports arouse little interest partly because of the complications and the limitations on high-risk patients. Later, the less invasive pituitary neuroadenolysis were devised using the transsphenoidal route. Since the advent of stereotactic radiosurgery targeting pituitary, small-scale clinical trials have been conducted in multiple centers worldwide. During the historical evolution, complications were reduced dramatically while the high efficacy was maintained.

Pituitary radiosurgery is a potential supplement of interventional treatment in patients with refractory malignant pain, although a larger and more homogenous sample is desired.

Here, we present a literature review of hypophysectomy of all three stages for the treatment of refractory cancer pain and the hypotheses of the mechanism. We also conclude the remaining questions not explained by the existing hypotheses and discuss the possible role of the hypothalamic–pituitary axis.

## 2 Methods

To investigate the historical evolvement of hypophysectomy, pituitary neuroadenolysis and pituitary radiosurgery and the possible mechanisms, we conducted a literature search using PubMed and make a supplement through manual searching. We used a combination of keywords and phrases, including (1) hypophysectomy, (2) pituitary neuroadenolysis or chemical hypophysecotmy, (3) pituitary radiosurgery or gamma knife hypophysectomy, (4) cancer pain. Inclusion criteria were: (1) reports on hypophysectomy, pituitary neuroadenolysis(including alcohol-induced, cryohypophysectomy, radio-active implantation) or pituitary radiotherapy (including gamma knife and cyber knife) for the treatment of cancer-related pain (without limitation of cancer types); (2) reports on clinical outcomes of pain relief and/ or complications; (3) written in English language. Exclusion criteria were applied to filter out sources that did not meet the defined criteria or were not directly relevant to the review topic.

## 3 Results

### 3.1 History

#### 3.1.1 Surgical hypophysectomy phase

Luft and Olivecrona first described pain relief after hypophysectomy in patients with malignant tumors related to hormonal milieu (7). This tactic was meant to suppress metastatic tumor growth related to a depressing action on the hormone production of the pituitary since it was a logical extension of hormonal manipulation by gonadectomy or adrenalectomy (8). Later, it was found that hypophysectomy as well as the antecedent operations produced pain relief effects more consistently than objective tumor regression. Transcranial approaches were replaced by transsphenoidal routes because of their appreciable morbidity and incomplete resection resulting from difficult access (7, 9–11).

The overall clinical results of initial efficacy for pain relief from advanced cancer after surgical hypophysectomy through both transcranial routes and transsphenoidal routes among 117 patients was 85.5% (ranging from 70.6 to 90.7%), the long-term efficacy was 85.1% (ranging from 64.7 to 92.6%) (Table 1). The most frequent adverse event is diabetes insipidus(DI) and hypopituitarism resulting from the removal of the pituitary. Others include cerebral spinal fluid(CSF) leak, damage to the optic and both olfactory nerves, intracranial clot need reoperation(1–6%), meningitis, acute cortisone deficiency, nasal infection, crusting and deaths caused by a cerebral hemorrhage and brain softening resulting from the transcranial route (Table 1) (10–14).

**Table 1:** Pain relief outcomes of hypophysectomy and pituitary neuroadenolysis hypophysectomy.

Study	No. of patients	Procedure	Initial pain results		Long-term pain results		Adverse events
			Effects	Efficacy	Effects	Efficacy	
Transcranial							
1953, Luft et al.	12	Transcranial Hypophysectomy	2 with pain relief, others without quantification of pain improvement	–	1/2 recurrence	–	No deaths or serious complications of any kind
1959, Cobb et al.	19	Transcranial Hypophysectomy	19/19 with striking relief of pain	100%	not described	–	Not described
1962, Scott et al.*	–	Transcranial Hypophysectomy	–	–	–	–	–
1974, Thompson et al.*	–	Transcranial Hypophysectomy	–	–	–	–	–
Transphenoidal							
1969, Kapur et al.	63	Transphenoidal Hypophysectomy	No quantification of pain improvement	–	–	–	DI (11, 17.5%), CSF rhinorrhea (4, 6.3%), deaths(6, 9.5%), hemorrhage (1, 1.6%), meningitis (1, 1.6%), infection (1, 1.6%), cortisol deficiency (3, 4.8%)
1971, Hardy et al., *	All technique, no results	Transphenoidal Hypophysectomy	–				–
1975, Gros et al.*	–	–	–	–	–	–	–
1977, Tindall et al.	6	Transphenoidal Hypophysectomy	5/6 with pain relief	83.3%	1/5 recurrence	80.0%	Partial DI (4, 66.7%), no other complications
1977, Silverberg et al.	17	Transphenoidal Hypophysectomy	12/17 with subjective improvement (improvement in bone pain, increase in appetite, weight gain, and an increased sense of well-being)	70.6%	5 patients with remission: duration of 11 months (range: 5–20); 7 patients without remission of 3.8 months (range: 1–6)	–	DI (6, 37%), CSF rhinorrhea (1, 16.7%), infection and meningitis (1, 16.7%)
1979, Tindall et al.	53	Transphenoidal Hypophysectomy	39/43 with pain relief, mean relief 2.2 mo	90.7%	6 to 18 mo survival: 19/21 with pain relief, mean relief 6.6 mo; > 18 mo survival: 6/6 with pain relief, mean relief 11.0 mo	92.6%	DI (40, 75.5%; 32, permanent; 8, temporary), CSF rhinorrhea (6, 11.3%), deaths (4, 7.5%)
1981, Schwarz et al.	45	Transphenoidal Hypophysectomy	36/45 with pain relief	80.0%	The duration of pain relief was 8 mo (mean) in group 1, 5 mo in group 2, and < 2 mo in the nonresponding group * group 1: with objective response (at least 25% shrinkage or cessation of growth of known lesions and no new lesions) for more than 6 mo * group 2: with objective response lasting 3 to 6 mo	–	DI (41, 92%; 3 require vasopressin), CSF rhinorrhea (3, 6.7%, early cases), nonfatal bacterial meningitis(1, 2.2%)
1983, Takeda et al.	17	Transphenoidal Hypophysectomy	15/17 complete	88.2%	4/15 recurrence	64.7%	DI (16/18, 88.9%), CSF rhinorrhea (4/18, 22.2%), meningitis (2/18, 11.1%), hemorrhage and death (1/18, 5.6%); transient euphuria (‘common’), hyperthermia (4/18, 22.2%), hallucinations (4/18, 22.2%)
1984, Smith Jr. et al.	15	Transphenoidal Hypophysectomy	11/15 improvement of pain	73.3%	7/15 appreciable pain relief for >2 mo(mean 7 months, 4 to 16 mo)	46.7%	Not described
				84.6%		75.8%	
Neuroadenolysis of the pituitary alcohol adenolysis							
1957, Greco et al. *#	–	Alcohol adenolysis	–	–	–	–	–
1965, Greco et al. *#	–	Alcohol adenolysis	–	–	–	–	–
1976, Moricca et al.*	–	Alcohol adenolysis	–	–	–	–	–
1977, Corssen et al.	24	Alcohol adenolysis	13/24 complete +10/24 improvement; after second NALP: 1/24 complete	100.0%	13/24 complete	54.2%	DI, ptosis and mydriasis (1, 4.2%), headaches, nosebleed (1, 4.2%)
1977, Katz et al.	13	Alcohol adenolysis	11/13 good to excellent pain relief	84.6%	–	–	DI need treatment (10, 76.9%), extraocular nerve palsies (3)
1978, Levin et al.	10	Alcohol adenolysis	9/10 good to excellent pain relief	90.0%	9/10 good to excellent pain relief	90.0%	Significant DI (8, 80.0%), transient CSF rhinorrhea (2, 20.0%), CN3 palsy (1, 10.0%), bitemporal hemianopia (1, 10.0%)
1979, Katz et al.	27	Alcohol adenolysis	26/27 good to excellent pain relief	96.3%	26/27 good to excellent pain relief	96.3%	Ocular-nerve palsies (2, 7.4%)
1978, Lipton et al.	92	Alcohol adenolysis	38/92 complete +28/92 partial	71.7%	20%(18/92) complete pain relief for at least 4 mo	20.0%	DI, CSF rhinorrhoea (5, 5.4%), death (4, 4.3%), temporary dilatation of a pupil (5, 5.4%), hemorrhage (3, 3.3%, 1 probably contributed to a demise)
1979, Madrid et al.*	–	Alcohol adenolysis	–	–	–	–	–
1979, Miles et al.*	–	Alcohol adenolysis	–	–	–	–	–
1980, Levin et al.	29	Alcohol adenolysis	27/29 good to excellent pain relief	93.1%	25/29(1 underwent a repeat injection, 1 underwent a cordotomy)	86.2%	DI, CSF rhinorrhea (1, 3.4%), CN3 palsy (4, 13.8%), visual defect (3, 10.3%)
1980, Williams et al.	11	Alcohol adenolysis	5/11 complete(2 after second injection), 3/11 moderate(1 after second injection)	72.7%	5/11 complete(2 after second injection), 3/11 moderate(1 after second injection)	72.7%	DI (2, 18.2%)
1981, Loyd et al.	34	Alcohol adenolysis	25/34 good analgesia	73.5%	Not described	–	DI (6, 17.6%), CSF rhinorrhea (2, 5.9%), hemorrhage (1, 2.9%, abandoned procedure), headache (6, 17.6%), vomiting/nausea (3, 8.8%), visual defects (1, 2.9%), pupil change (3, 8.8%, temporary), epistaxis (1, 2.9%), myxoedema (1, 2.9%)
1983, Takeda et al.	102	Alcohol adenolysis	83/101 complete(2 after second NALP) + 10/101 incomplete(5 after second NALP)	92.1%	69/101 complete(16 after second NALP, 4 after third NALP without description of efficacy) + 12/101 incomplete(2 after second NALP);	80.2%	DI (69/136, 50.7%), CSF rhinorrhea (1/136, 0.7%), meningitis (2/136, 1.5%), visual defects (10/136, 7.4%), transient ophthalmoplegis (4/136, 2.9%), temporary headaches (50/136,36.8%); transient euphuria (‘common’), hyperthermia (‘rare’), hallucinations (‘few’)
1987, Waldman et al.	15	Alcohol adenolysis	9 no more narcotic analgesics +5 oral narcotic analgesics+1 incomplete pain control	–	–	–	Transient diplopia (1)
				84.8%		80.2%	
Cryohypophysectomy							
1964, Rand et al.*	–	Cryohypophysectomy	–	–	–	–	–
1971, Maddy et al.	20	Cryohypophysectomy	7/19 with remissions +5/19 without remissions	63.2%	1 recurrence	57.9%	Transient DI (7, 35.0%), CSF rhinorrhea (3, 15.0%), death (1, 5.0%), transient hyponatremia (5, 25.0%)
1979, West et al.*	–	Cryohypophysectomy	–	–	–	–	–
1982, Avellanosa et al. *	–	Cryohypophysectomy	–	–	–	–	–
1983, Duthie et al.	18	Cryohypophysectomy	>50% pain relief, 1 week: 15/18; 1 mo:11/14; 2 mo: 5/7; 3 mo: 3/4;	83.3%	–	–	CSF rhinorrhea (4, 22.2%, transient), headache (8, 44.4%, severe but transient), slight blurring of vision (1, 5.5%)
				73.0%		–	
Radio-active implantation							
1959, Forrest et al.	45	Radio-active implantation(Y-90)	Pain not assessed	–	–	–	DI (‘common’), CSF rhinorrhea (2/45, 4.4%), vision loss (8/45, 17.8%); CN3 palsy (2/45, 4.4%), meningitis and death (1/45, 2.2%)
1962, Talairach et al.*#	–	Radio-active implantation(Y-90)	–				–
1969, Notter et al.*	–	Radio-active implantation(Y-90)	–	–	–	–	–
Other							
1969, Zervas et al.	164	Radiofrequency ablation	31% (of the 66) with pain improvement (without remission)				CSF rhinorrhea, meningitis
1969, Arslan et al.*	–	Ultrasonic selective hypophysectomy	–	–	–	–	–

*No original articles, # non-English articles. *DI, Diabetes Insipidus; CSF, Cerebral spinal fluid; NALP, Neuroadenolysis of the pituitar. The long-term effects are measured until the following terminal or the deaths.

#### 3.1.2 Pituitary neuroadenolysis phase

Pituitary neuroadenolysis, also called chemical hypophysectomy, is a less invasive procedure that can be repeated freely for pain recurrence or manifestations of tumor growth. Alcohol-induced adenolysis was the most widely used method in the 1970s, first performed by Greco and Moricca independently in 1957 and 1958. In a later presentation in 1975, Moricca reported an expanded series of 884 patients undergoing 2,120 procedures (15). Other methods include ultrasound destruction, cryoablation, thermocoagulation, radiotherapy, external irradiation with heavy particles (especially alpha rays and protons), and direct implantation of radioactive substances, such as seeds or pellets of ^90^Y, ^198^Au, and ^32^P, suffering from the possibility of incomplete ablation (9, 16–19). Subsequent improvements include Moricca’s larger volumes of alcohol, Corssen’s decrease of needles used and Levin and Katz’s introduction of the stereotactic head frame (9, 15, 20).

The overall clinical results for pain relief from advanced cancer after neuroadenolysis among 397 patients was 75.06% (Table 1). The main complication after neuroadenolysis is transient diabetes insipidus (40%) and hypopituitarism (15%) resulting from the pituitary destruction (21). Complications resulting from the transsphenoidal route include disturbances of surrounding tissues(mostly transient), headache, rhinorrhea/CSF leak(20%), and hemorrhage (9, 22, 23). Severe complications include infections such as meningitis, and death (Table 1) (22, 24).

#### 3.1.3 Pituitary radiosurgery phase

Radiosurgery was first employed to treat refractory cancer pain targeting the centromedian thalamic nucleus and other terminal thalamic endpoints for the paleospinothalamic tract fibers (25–31). The pituitary is then proved to be a superior target (32). Pituitary radiosurgery is also referred to as gamma (knife) hypophysectomy, first applied in this field by Backlund in 1972 as an improved method of radioactive ablation (33). Following Leksell’s study in thalamotomy, Backlund et al. administered 200 to 250 Gy doses targeting at the anterior two-thirds of the pituitary, which had similar efficacy to later studies with improved MRI and CT imaging that used 160 Gy (24, 33–38).

After 30 years, Hayashi et al. used Gamma knife surgery targeting the stalk of the pituitary gland with doses of 150–200 Gy (24). The patients have been followed for longer periods, filling in the blanks of long-term effects after this procedure. Few adverse effects have been reported in all the studies produced by different centers with the same targeting zone and doses, while those targeting the gland reported higher risks of diabetes insipidus and hypocortisolism (24, 34–37).

Lovo et al. placed the higher isodose lines in the most posterior part of the neurohypophysis, which may be a possible reason for the lower initial effects and higher rates of recurrence. In 2022, Lovo et al. induced a triple target irradiation in the hypophysis and bilateral thalamus, with a lower max dose of 90 Gy at each target, as a treatment alternative for the refractory oncological pain of mixed nature (nociceptive, neuropathic, and visceral).

The overall clinical results for initial complete pain relief after pituitary radiosurgery among 64 patients was 95.9% (ranging from 80.0 to 100.0%), for the long-term, was 73.5% (ranging from 30.0 to 100.0%) (Table 2). Backlund’s original study reported frequent diabetes insipidus and hormonal defects, which may be associated with the high dose ranging from 200 to 250 Gy (33). Hayashi’s series of studies reported no complications, while subsequent studies using the same parameters only reported individual cases of complications such as diabetes insipidus and hormone reduction (24, 35) (Table 2).

**Table 2:** Pain relief outcomes of pituitary radiosurgery.

Study	No. of patients	Type of pain	Method of delivery	Radiosurgical target	Max dose (Gy)	Pain results/initial effects	Initial efficacy	Long-term effects	Long-term efficacy	Adverse events
1972, Backlund et al.	8	Bone metastases	2–3 isocenters, 3 × 5 or 3 mm × 7 mm cross-sectional beams	Anterior two-thirds of the pituitary	200 or 250	4/4 survivors pain relief	100.0%	Not accessed	–	Hormonal deficiencies (8, 100%), DI(‘frequent’)
1998, Liscák et al.*#	1	–	–	Pituitary gland	150	Complete pain relief lasted for 24 months after SRS	–	–	–	Hypocortisolism
2002, Hayashi et al.	9	Bone metastases	1 isocenter, 8 mm × 4 mm patients;2 isocenters, 4 mm × 5 mm patients	Junction between the pituitary gland and stalk	150–200	9/9 pain free within a few days	100.0%	9/9 pain free (permanently)	100.0%	None
2003, Hayashi et al.	6	Bone metastases	1 isocenter, 8 mm collimator	Junction between the pituitary gland and stalk	160	6/6 pain free within a few days	100.0%	6/6 pain free (1–4 mo)	100.0%	None
2004, Kwon et al.	7	Cancer metastases	1 isocenter 8 mm × 3 mm patients;2 isocenters 4 mm × 4 mm patients	Junction between the pituitary gland and stalk	150–160	7/7 pain relief(>50%)	100.0%	5/7 pain relief(without relapse)	71.4%	DI/hypopituitarism (1, 14.3%)
2019, Lovo et al.	11	Bone metastases or other organs affected	1 isocenter, 8 mm collimator	Neurohypophysis	150	4/10 minimal to no pain, 3/10 managed with medications, 1/10 < 50%, 2/10 no response within 2–5 days	80.0%	3/10 pain relief, 2/10 reccurence, 2/10 no response	30.0%	None
2020, Golanov et al.*#	1	–	–	Junction between the pituitary gland and stalk	150	The maximal analgesic effect was reached on the 5th day after SRS and was permanent. Significant reductions in analgesic doses and prominent improvements in quality of life were noted	–	–	–	None
2022, May et al.	20	Bone metastases	1 isocenter, 8 mm collimator (2 segments modified to 4 mm)	Pituitary gland	150–200	10/10 pain relief within 2 to 4 weeks	100.0%	10/10 pain relief(>50%, average 24% remaining pain, permanently)	100.0%	DI and hypocortisolism(3, 6.0%), transient abducens nerve palsy(1)
2022, Lovo et al.	3	Mixed pain (nociceptive, neuropathic, and visceral)	1 isocenter at hypophysis, 8(GK)/7.5(CK) mm collimator, 2 isocenters at bilateral thalamus, 4(GK)/5(CK) mm collimator	Hypophysis, and mesial structures of the bilateral thalamus	90	3/3 pain relief within a few days	100.0%	3/3 pain relief (3 to 5 weeks)	100.0%	Excessive sleepiness(1)
Total number of patients	64						47		36	
							95.9%		73.5%	

*No original articles, # non-English articles. *DI, Diabetes Insipidus; CSF, Cerebral spinal fluid; GK, Gamma Knife; CK, Cyber Knife.

**Figure 1:**
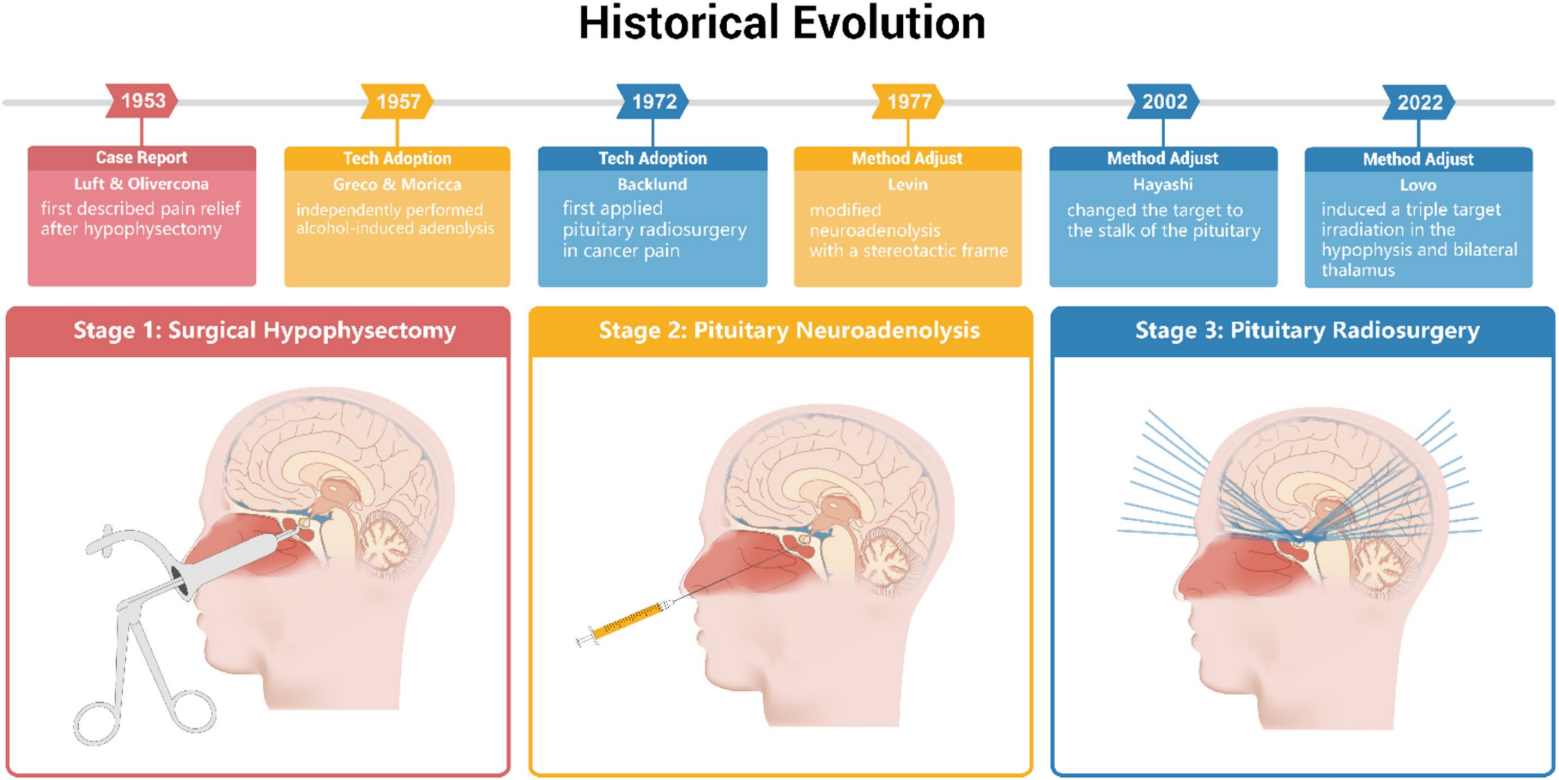
Historical evolution of hypophysectomy, pituitary neuroadenolysis and pituitary radiosurgery. The timeline of case report, technique adoption and method adjustion in the three stages of hypophysectomy. Stage 1, surgical hypophysectomy (red), stage 2, pituitary neuroadenolysis(yellow) and stage 3, pituitary radiosurgery(blue). This figure is created using Adobe Illustrator.

Table 3 summarizes the clinical outcomes and rate of complications after hypophysectomy of all three stages (Figure 1).

**Table 3:** Summary of efficacy and safety after hypophysectomy by different treatment modalities.

	Surgical hypophysectomy	Neuroadenolysis			Radiosurgery
		Alcohol-induced	Cryoablation*	Radiation/brachytech*	
Efficacy					
Initial pain results	84.6%	84.8%	73.0%	-	95.9%
Long-term pain results	75.8%	80.2%	-	-	73.5%
Safety					
DI	17.5–92.0%	17.6–80.0%	-	‘common’	0–14.3%
CSF rhinorrhea	0–22.2%	0.7–20.0%	-	4.4%	0%

*1 article for cryoablation and 2 for radiation/brachytech.

### 3.2 Mechanism hypotheses

**Figure 2:**
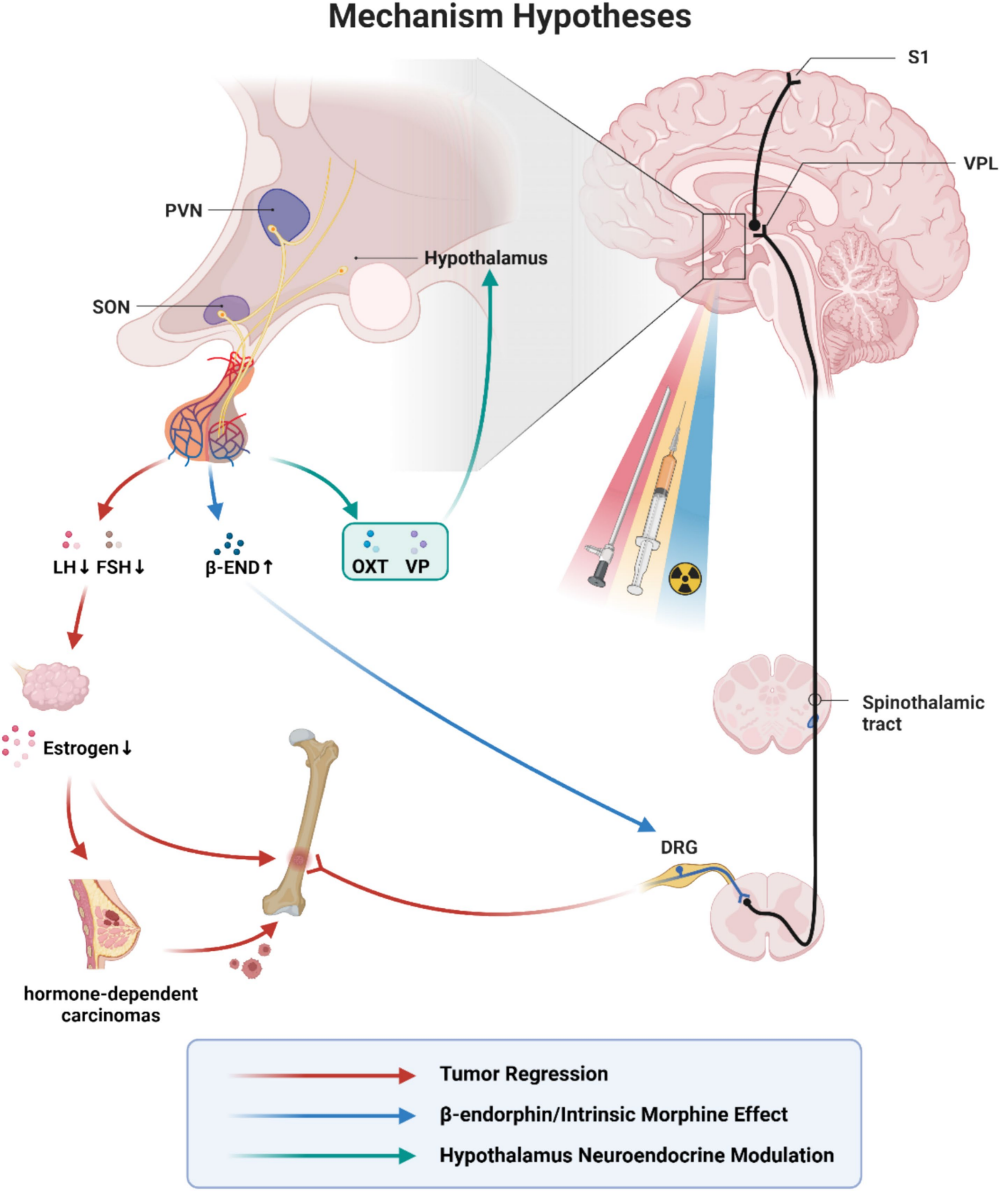
Mechanism hypotheses of hypophysectomy, pituitary neuroadenolysis and pituitary radiosurgery. VPL, ventral posterolateral nucleus; PVN, paraventricular nucleus of hypothalamus; SON, supraoptic nucleus; LH, luteinizing hormone; FSH, follicle stimulating hormone; *β*-END, β-endorphin; OXT, oxytocin; VP, vasopressin; DRG, dorsal root ganglia. The similar efficacy and different complications due to the routes and the techniques indicate the same key problem lying behind hypophysectomy, pituitary neuroadenolysis and pituitary radiosurgery. The existing mechanism hypotheses mainly include: (1) Tumor regression(red). Hypophysectomy was originally conceived as a means of achieving hormone-dependent tumor regression. The earliest theories hypothesized that tumor regression contributed to pain reduction. (2) Intrinsic morphine-like effect(blue). β-endorphin is a kind of endogenous opioid receptor agonist and plays a role in mediating opioid-dependent analgesia in acute pain. Its precursor localizes in the pituitary gland and the arcuate nucleus in the hypothalamus. It has been suggested that the removal of the pituitary may lead to a compensatory overproduction of β-endorphins precursors into the blood and cerebrospinal fluid. There was a temporary sharp increase in CSF β-endorphins immediately after neuroadenolysis, but returned to baseline on the third day after surgery. (3) Hypothalamus neuroendocrine modulation (green). The hypothalamic–pituitary axis is considered to exert a long-lasting suppressive effect on the mediation and perception of cancer pain through C-fibres and the central nervous system, while the specific working hormones remain unknown. It has been suggested that pain relief may result from the modulation of central pain-inhibiting neurons through a humoral agent distributed by the cerebrospinal fluid or a direct neural stimulus. With the recent exploration of the analgesic effects of oxytocin and vasopressin, it is possible that these two hormones may play a role. This figure is under copyright with our institution, so we have submitted a license for the CNS to publish it. With permission from ©BioRender.com, with permission. All rights and ownership of BioRender content are reserved by BioRender.

The mechanisms of surgical and chemical hypophysectomy have proved to be fundamentally similar, but whether pituitary radiosurgery worked via the same mechanism is unknown. The three stages all appeared as modified procedures of the previous stage, with similar efficacy and different complications due to the routes and the techniques. This indicates the same key problem lying behind them (Figure 2).

#### 3.2.1 Tumor regression

Hypophysectomy was originally conceived as a means of achieving hormone-sensitive tumor regression as a logical extension of hormonal manipulation by gonadectomy or adrenalectomy. The earliest theories hypothesized that tumor regression contributed to pain reduction (15, 39). Takeda et al. demonstrated that neuroadenolysis resulted in tumor regression in 6.9% of cases with hormone-dependent carcinomas, while surgical hypophysectomy in 55.5% of cases. 88% of cases obtained pain relief (13). Hayashi et al. reported that the original cancer did not change in size, but the tumor markers transiently decreased 2 weeks after pituitary radiosurgery (24).

Various lines of evidence challenge the notion: (1) pain relief occurs in cases of thalamic pain and malignancies not hormone-dependent (9, 12, 40–42); (2) some patients with breast and prostate carcinoma are unresponsive to hormone manipulation (10, 12, 42, 43); (3) patients may obtain relief despite a failure to obtain an objective remission (10–12, 19, 43). Levin et al. reported that 40 to 60% of the patients who obtain pain relief ultimately show no obvious tumor regression, suggesting that the hormonal sensitivity of the tumor may not be an adequate predictor of the potential for achieving pain relief either. However, they also mentioned the possibility that slight degrees of regression undetectable by roentgenogram may contribute to the relief. In conclusion, tumor regression may serve to complement pain relief, while the two processes may be independent (44).

#### 3.2.2 *β*-Endorphins/intrinsic morphine-like effect

β-endorphin is a kind of endogenous MOPr(*μ*-opioid receptors) agonist and plays a role in mediating opioid-dependent analgesia in acute pain through hyperpolarizing effects and suppression of neuronal activity (45). Its precursor localizes in the pituitary gland and the arcuate nucleus in the hypothalamus. It has been suggested that the removal of the pituitary may lead to a compensatory overproduction of *β*-endorphins precursors into the blood and cerebrospinal fluid (35, 36, 46). Takeda et al. demonstrated that there was a temporary sharp increase in CSF β-endorphins immediately after pituitary neuroadenolysis, but returned to baseline on the third day (47). While Deshpande et al. suggested that patients treated by alcohol-induced neuroadenolysis were still capable of secreting β-endorphin after more than 4 years (48). Endorphins may play a role in immediate pain relief after surgery but are not the sole cause of long-lasting analgesia (13, 20, 44, 46, 47). Some naloxone studies have shown positive results to reverse the pain-relieving effects, while others are the opposite. The failure of pain restoration does not necessarily exclude an opiate-mediated effect, considering of different classes of opiate receptors with varying affinities for naloxone (44, 49, 50). Naloxone insensitivity could be due to limited affinity rather than indicating a non-opioid mechanism. The reversal effect depends on the degree of pituitary destruction as well (46).

#### 3.2.3 Neuroendocrine modulation

Several lines of evidence suggest that pain relief from hypophysectomy is not directly related to the expected decrease in levels of pituitary hormones (44). Instead, pain relief is believed to result from the modulation of central pain-inhibiting neurons through a humoral agent distributed by the cerebrospinal fluid or a direct neural stimulus. Increase of the peptides synthesized in the hypothalamic–pituitary axis and suppression of pituitary function are considered to exert a long-lasting suppressive effect on the mediation and perception of cancer pain through C-fibres and the central nervous system (11, 51).

##### 3.2.3.1 Hormones

The specific working hormones remain unknown. Takeda et al. discussed the alterations of hypothalamic–pituitary axis action in detail and examined pituitary hormones including insulin, TRH, gonadotropins (LH, FSH), HGH, TSH, prolactin (PRL), ACTH, and vasopressin (47). The anterior pituitary hormones commonly decrease to varying degrees after surgery except ACTH. 85.7% of the cases with slight/moderate suppression of anterior pituitary function showed complete pain relief, while 90.9% in the markedly suppressed group. These pieces of evidence suggest a negative relationship between anterior pituitary function and pain relief. Both endorphins and ACTH have a common precursor, but in higher primates and man, the synthesis of these two peptides is regulated by separate mechanisms (48, 52). ACTH shows a significant increase in the early postoperative stage and is even more pronounced in CSF compared to blood, even 2 months later in cases with complete pain relief, in contrast to the minimal elevation observed in cases without complete relief. TRH in CSF exhibited an abrupt increase after ablation, followed by a subsequent decline to a level slightly higher than the preoperative level. It then gradually increased again, demonstrating a fluctuating pattern. TRH was consistently elevated 24 h after surgery and continued to rise in all cases with pain relief. Vasopressin also showed an abrupt elevation in the CSF immediately after surgery, followed by a decrease, but remained at a level higher than the preoperative level even 3 weeks later. In contrast, the levels of vasopressin in serum did not show a significant increase and were consistently lower than those in the CSF (47). Lovo et al. proposed that the oxytocin is redirected when a high dose of radiation is administered to the hypophysis, and termed it the radio-endocrine-modulatory effect (53).

##### 3.2.3.2 Pain fiber pathways

Some scholars found that the efficacy of hypophysectomy remains the same regardless of the extent of pituitary gland destruction and may be caused by reactionary hyperactivity of the hypophyseal system exerting inhibitory influences on the pain pathways of the brain (46). Contrast media injected into the hypophysis spread beyond and destroyed the hypothalamus and adjacent structures (7, 40, 43, 46, 54, 55). These pieces of evidence indicate that hypophysectomy may interfere with neural pathways and result in the impairment of pain conduction and the ability to interpret pain properly (46).

Ample anatomic evidence shows extensive neural connections among the hypothalamus, thalamus, limbic system, and various portions of the central gray matter. These systems receive information from lower spinal areas such as the lateral spinothalamic tract (15). Stereotactic electrical stimulation of these areas in animals has been proven to decrease responses to noxious stimuli by enhancing mechanisms that modify pain appreciation and result in analgesia (56–60). Periventricular and central gray regions, in particular, are drought to produce a morphine-like substance that decreases the response to aversive stimuli (61). Takeda et al. indicate that the peptides mainly synthesized in the hypothalamic–pituitary axis would increase in the CSF after hypophysectomy, and suppress the mediation and perception of cancer pain through the C-fibers and the central nervous system (47).

It has been proven that peripheral pain receptor sensitivity is not related since hypophysectomy does not alter the normal sensitivity to pinprick or acute injury pain (62, 63).

#### 3.2.4 Hypothalamus

Some investigators have proposed that concomitant damage to the hypothalamus plays a critical role in producing pain relief (15, 40, 44, 62). Lipton et al. discovered that contrast medium could spread above the sella, ascend the pituitary stalk, and eventually breaking through into the third ventricle (64). Postmortem examinations conducted by Levin revealed subependymal gliosis along the floor of the third ventricle, significant cell loss in the supraoptic and paraventricular nuclei, and damage to the median eminence (40). These findings suggest that pain relief following alcohol injection may occur through the destruction of thalamic and hypothalamic nerve pathways and interfere with pain conduction or may impair the patient’s ability to interpret pain properly. However, further pathological studies are needed to confirm this (9, 20). Other investigators have demonstrated that lesions in the posterior inferior peri-third ventricular area produced good relief in over 70% of patients with pain caused by malignant tumors, highlighting the importance of the posteromedial hypothalamus in pain control (44).

Later, Levin et al. proposed that the pain relief following hypophysectomy may be more directly related to the stimulation of hypothalamic function. Oophorectomy, adrenalectomy, and orchiectomy have all been found to produce prompt pain relief within hours after surgery in patients with metastatic breast or prostate carcinoma, even before objective remission occurs. The time of onset of pain relief seems to be similar to that after hypophysectomy. These observations suggest the existence of a common mechanism of pain relief for all four operations, possibly involving a hypothalamic pain-suppressing response that is triggered by the elimination of hormonal feedback (44).

Takeda and other researchers reported that, following neuroadenolysis, most patients experienced clinical manifestations of hypothalamic involvement, including temporary euphoric states, increased appetites, hypothermia, and hallucinations almost 2 days later, which continued for several days (13, 23, 47). In Hayashi’s series of studies, they confirmed these clinical symptoms and discovered that MR spectroscopy demonstrated a stimulating effect in the hypothalamus, with a significant increase in the level of N-acetyl aspartate within 24 h after radiosurgery operations (24, 35). Nevertheless, the precise role of the hypothalamus in the pain-relief mechanism remains unclear.

## 4 Discussion

### 4.1 Remaining questions

#### 4.1.1 Onset time and duration of pain relief

Pain relief typically begins a few hours after hypophysectomy and lasts for several months, none of the theories can explain both the immediate and long-term effects (24, 34–38). This suggests that different mechanisms may be responsible for each stage, with a possible overlap between them. We notice that in previous studies, the VAS scores typically change in the following mode: a sustained decline immediately after the operation, then rebounds and eventually a stablization around 20–30 days. The restable point may be the alteration of the short-term and long-term mechanisms (37, 53).

Two hypotheses may explain the immediate pain relief after the operation: endorphins and neuro-endocrine modulation. The role of endorphins remains unclear because some naloxone studies failed to reverse the pain-relieving effects. The rapid relief after operations indicates that it is mediated by the central nervous system rather than serum hormone concentration, but the specific hormones responsible for the pain relief effects are unknown either. Tumor regression is the only explanation for long-term pain relief, but pain relief continues even when the tumor progresses and normal pituitary function returns.

#### 4.1.2 Type of pain

Pituitary radiosurgery is applied in poststroke thalamic pain using the same doses, indicating a central rather than a systemic mechanism. 71 to 76.5% of patients had initial pain reduction within 48 h, but only 21 to 38.5% had long-term effects (43, 52). Similar initial efficacy to cancer pain and different recurrence rates indicate separate mechanisms between the two phases. Projections from the paraventricular nucleus (PVN) innervate lamina I of the spinal dorsal horn, peri mesencephalic gray, and the nucleus raphe magnus are important pain-modulating centers and the PVN may be the key anatomic locus for pain control (44).

Some scholars reported that patients with bone metastases have the best response to hypophysectomy (10, 11, 19). Jessiman et al. stated that they have seen osseous secondaries heal while soft tissue deposits enlarge. Some scholars suggested that the site of secondaries would not determine the response. Cade supposed that metastasis behaves according to the ‘all or none phenomenon’; if one group of metastases responds then all others would do the same (44).

#### 4.1.3 Pain assessment

There are some fundamental issues in pain assessment as well. First, there is no standardized definition of pain relief. Most studies employ pain rating scales, the quantity of analgesics or a combination. Second, the division of the short-term and long-term effects is unclear, and it should be noted that the survival times of cancer patients are hugely different. Third, the assessment is rarely based on the reports of independent observers. Last, few studies provide a careful account of the cause of pain in each patient (44).

#### 4.1.4 Target area

Neurosurgical interventions mainly interrupt neural circuits involved in pain processing or modulation to relieve intractable pain. Thalamotomy targeted at the nuclei of the medial thalamus, which relays information related to the affective motivational (unpleasantness) dimension of pain. Only 51% of patients had initial pain relief and 35% had long-term effects during the follow-up periods, which is hugely different from hypophysectomy (65).

In radiosurgery hypophysectomy, different substructures of the pituitary have been targeted. Hayashi et al. targeted the junction of the pituitary gland and stalk, while Lovo et al. changed it to the neurohypophysis and failed (24, 35–37, 39). 20% of patients had no response, and 50% of patients presented recurrent pain at the end of life (37). Later, the target was changed to the hypophysis and mesial structures of the bilateral thalamus irradiating with a very small maximum dose of 90 Gy (53). They speculated that single-target radiation could only reduce pain but not eliminate it, and most cases would recur especially before death (53, 66).

#### 4.1.5 Radiation dose

The mechanism behind surgical and chemical hypophysectomy is the destruction of substructures of the pituitary. The function of radiosurgery is now thought to be not only radiosurgical ablation but also radiomodulation, even radio-endocrine-modulation. It is still unknown whether the relief of cancer pain is due to damage or modulation, while the dose setting of gamma knife surgery is still empirically based and the optimal radiation dose is yet not determined.

In the triple target irradiation study of Lovo et al., they delivered only 90 Gy to each region instead of 150–200 Gy, a single target in other similar studies. However, they still made the pain more bearable and responded better to medication. Their experience also showed that these treatment strategies, either single, dual, or triple target irradiation, eliminate pain in most oncological patients. Still, they allowed unmanageable intense pain to be more bearable and to respond better to basal pain medication (53).

According to Hayashi et al., there was no evidence of destructive changes, no dysfunction of endocrinological status, and no morphological changes on follow-up MR images. Clinical symptoms and MR spectroscopy revealed a stimulating effect on the hypothalamus (24, 35).

### 4.2 A new hypothesis—role of hypothalamic–pituitary axis

Some researchers suggested radio-endocrine modulation of the hypothalamus as the cause of pain relief effect, rather than pituitary destruction (24, 35, 46). We believe the mechanism may be similar to the triphasic diabetes insipidus after the pituitary stalk injury during neurosurgical operations. In the early stages, due to the blockage of the pituitary stalk, some hormones stored in the hypothalamus are nowhere to be released and thus flow into the cerebrospinal fluid and modulate the central pain-inhibiting neurons—the sustained decrease of VAS scores in the few days after operations approve the hypothesis. The anterior pituitary function has been reported to not correlate with the analgesic effect, but that of the posterior pituitary is controversial. Some scholars found a significant association between the post-hypophysectomy response and diuresis (a symptom of posterior pituitary function loss), while others disagreed (10, 54, 67, 68). We posit that patients suffering from DI after hypophysectomy certainly have pain relief effects at the same time, but there is no relevance between them (Table 4). Partial blockage of the pituitary stalk can lead to an analgesic effect without the occurrence of diabetes insipidus. It is postulated that only a few supraoptic nuclei need to remain to prevent polyuria (69). The working hormones may be vasopressin and oxytocin. They have common structures and can be traced back to the same ancestor (70). Oxytocin can directly and indirectly modulate pain through both the central and peripheral nervous systems (71). It has been observed that chronic pain reduction happened following oxytocin administration in humans, but it is not widely employed in pain management due to the short half-life and lack of specificity. Some evidence also indicates the role for vasopressin in pain (72). Vasopressin sharply increases in the cerebrospinal fluid several times within days after operations, consistent with the instantaneous pain relief effect. Then it gradually decreases to a level higher than pre-operation status, consistent with the longer effect. Radiosurgery hypophysectomy may redirect oxytocin toward hypothalamic regions affecting pain modulation (73). As the stored hormones are gradually consumed, the long-term mechanism begins to take effect, which we regard as the regeneration of the hypothalamus. Studies in craniopharyngioma have illustrated the remarkable regenerative capacity of neurohypophysis as early as 3 weeks after injury in animals. In humans, degeneration and regeneration of the hypothalamic nuclei are modest and happen at 3 days to 32 months after surgery.

**Table 4:** Diabetes insipidus and pain relief.

Study	No. of Patients	Procedure	Relation of DI and initial pain relief		
			DI and pain relief	Pain relief without DI	DI without pain relief
1969, Kapur et al.	63	Transphenoidal hypophysectomy	27/57	2/57	18/57
1979, Levin et al.	29	Alcohol adenolysis	25/29	2/29	1/29
1980, Williams et al.	11	Alcohol adenolysis	2/10	6/10	0/10
2002, Hayashi et al.	9	Pituitary radiosurgery	0/9	9/9	0/9
2003, Hayashi et al.	6	Pituitary radiosurgery	0/6	6/6	0/6
2004, Kwon et al.	7	Pituitary radiosurgery	1/7	6/7	0/7
1978, Lipton et al.	92	Alcohol adenolysis	No relationship between the DI and the degree of pain relief, some patients had complete pain relief with no DI		
1983, Takeda et al.	102	Alcohol adenolysis	No significant difference in cases with and without polyuria Obtainability of complete pain relief		

## 5 Conclusion

From surgical hypophysectomy to pituitary neuroadenolysis and pituitary radiosurgery, these procedures have shown similar pain relief effects, but fewer complications. Hypophysectomy is a promising treatment of refractory malignant pain, although a larger and more homogenous sample and a standardized treatment protocol are desired. Many items need to be standardized, including the criteria of pain relief, the instrument of pain assessment, and the range of short-term and long-term effects. Hypophysectomy could be an effective supplement of current pain management protocols. We are carrying out a related prospective, single-center clinical research program in Nanfang Hospital at present. Based on previous research, the immediate and long-term analgesic effects may not be attributed to a single mechanism. Several questions remain to be answered, including the contributing hormones in the neuro-endocrine modulation and the sustained mechanism. This would be a potential area for further work.
